# Efficacy of Intra-Articular Injection of Platelet-Rich Plasma Combined with Mesenchymal Stem Cells in the Treatment of Knee Osteoarthritis: A Systematic Review and Meta-Analysis

**DOI:** 10.1155/2022/2192474

**Published:** 2022-09-20

**Authors:** Weipeng Zeng, Gongze Wang, Xinping Liao, Caifeng Pei

**Affiliations:** ^1^Department of Joint Surgery, Hainan General Hospital (Hainan Affiliated Hospital of Hainan Medical University), Haikou 570311, China; ^2^Department of Sports Medicine, Qionghai People's Hospital, Qionghai 571400, China; ^3^Department of Orthopedics, People's Hospital of Wanning Hainan, Hainan 571500, China

## Abstract

**Objective:**

This study systematically evaluated the effect of intra-articular injection of platelet-rich plasma (PRP) and mesenchymal stem cells (MSC) on knee osteoarthritis (KOA).

**Methods:**

Randomized controlled trials (RCTs) of PRP combined with MSC in the treatment of KOA were collected from PubMed, Cochrane Library, Web of Science, Wiley online library, CNKI, and Wanfang databases from inception to July 30, 2022. Two researchers read and screened the literature to extract the data, respectively. After conducting a risk-of-bias assessment of included data, RevMan 5.3 software was used for meta-analysis. The Cochrane Handbook risk-of-bias assessment tool was used to evaluate the included literature.

**Results:**

A total of 9 papers with 480 KOA patients were included in this study. Significant differences in terms of visual analogue scale (VAS) score (MD = −1.10, 95% CI −1.91 to −0.29), *P* = 0.008) and knee injury and osteoarthritis outcome score (KOOS) (MD = 4.56, 95% CI 0.54–8.58, *P* = 0.050) were noted between the 2 groups. Subgroup analysis found that MD = 1.66 in KOOS-pain-1 group (95% CI (0.10, 3.22), *P* = 0.040), which is significant. The MD for KOOS total score and IKDC scores between the two groups was MD = 6.31 (95% CI 2.73–9.88, *P* = 0.0005) and MD = 3.05 (95% CI −7.09–13.20, *P* = 0.56), respectively.

**Conclusion:**

The results of this study provide a theoretical basis for the clinical treatment of KOA with PRP combined with MSC.

## 1. Introduction

Knee osteoarthritis (KOA) is a “joint whole organ disease” characterized by joint pain and loss of function related to intracellular homeostasis, loss of articular cartilage, injury of subchondral bone, and surrounding soft tissue [1, 2]. The prevalence of KOA increases with age and peaks in the middle-aged and elderly population. Furthermore, KOA accounts for the predominant cause of knee joint pain or even disability in middle-aged and elderly patients [3–5]. With the gradual increase of life expectancy of the population, the number of KOA occurrences is also increasing that brings a substantial economic burden to the public health and medical service. KOA has already become one of the main focuses for orthopedic surgeons with its high incidence [6, 7]. Although nonsteroidal anti-inflammatory drugs, hyaluronic acid (HA), and steroids are most widely used for the conservative treatment of KOA, the treatment efficacy is far from satisfactory. It has been established that these medications cannot halt disease progress nor repair the damaged cartilage [3]. Various biological agents have been shown to be promising for the treatment of KOA by promoting the healing of endogenous cartilage.

Platelet-rich plasma (PRP) has received extensive attention as a novel treatment for KOA. PRP is a form of regenerative medicine that maintains physiological balance by providing platelets, cell adhesion molecules, and glycoproteins. This method has been consistently confirmed to be effective for KOA by a host of reports [8–10]. However, PRP treatment is limited by short-term effects and compromised efficacy with increasing age [11]. Mesenchymal stem cells (MSC) have been noted to accelerate bone regeneration, enrich nutrient factor supply, and modulate immune reactions [12–14]. When PRP and MSCs are used in combination, the metabolic growth factors in PRP (such as fibroblast growth factor, transforming growth factor-*β*, epidermal growth factor, and insulin-like growth factor) and anti-inflammatory cytokines may have potential therapeutic effects for KOA [15].

This study utilized meta-analysis to integrate 9 published randomized controlled trials (RCTs) of PRP combined with MSCs that contained 480 patients for the treatment of KOA. This study aimed to provide a theoretical basis for the clinical treatment of KOA with RPP and MSC.

## 2. Materials and Methods

### 2.1. Database Retrieval

The articles on the treatment of KOA with PRP combined with MSC published from the establishment of the database to July 30, 2022, were searched on PubMed, Cochrane Library, Web of Science, Wiley online library, CNKI, and Wanfang database. The search terms were “platelet-rich plasma” OR “PRP,” “Mesenchymal stem cells” OR “MSCs,” and “knee osteoarthritis” OR “KOA.” The language of the literature is Chinese or English. The search strategies were as follows:

  PubMed
  #1 “platelet-rich plasma” OR “PRP”
  #2 “osteoarthritis” OR “Knee osteoarthritis” OR “KOA”
  #3 “Mesenchymal stem cells” OR “MSCs”
  #4 #1 AND #2 AND #3

### 2.2. Inclusion and Exclusion Criteria of the Literature

Inclusion criteria were as follows: (1) patients with primary KOA; (2) patients aged between 35 and 70 years regardless of gender; (3) Kellgren–Lawrence grades I–IV; and (4) the type of study was RCT. Exclusion criteria were as follows: (1) the patient received other medications within the past 6 months or used other drugs in the process of reintervention; (2) case reports, reviews, or inability to obtain the full text; (3) in the intervention group, only MSCs or PRP alone was used; and (4) duplicate publications. Two researchers independently screened and read the included literature. When disagreement emerged, a third researcher was consulted until an agreement was reached.

### 2.3. Literature Quality Evaluation

The quality of the literature was evaluated using the guidelines published in Cochrane handbook [16, 17], in which the evaluation indicators included the following: (1) selection bias (whether the included literature belongs to RCT); (2) group hiding (whether the group adopts blinding method); (3) blind method of both doctors and patients; (4) result evaluation blind method; (5) completeness of the report results; (6) publication bias; (7) other indicators in the paper including low risk, high risk, and unclear risk.

### 2.4. Statistical Analysis

Review Manager (RevMan) 5.3 was used for meta-analysis. The outcome indicators were analyzed with the standard mean difference (SMD) and 95% CI as effect size. The *I*^2^ and *P* tests were used for assessing the magnitude of heterogeneity. In the presence of *P* < 0.05 and *I*^2^ ≤ 50%, the included literature was considered to be homogeneous, and the fixed-effect model was used. Otherwise, the random-effect model was used. Subgroup analysis was performed to identify the source of significant heterogeneity. Publication bias was described by funnel plots and assessed using Egger's and Begg's tests. A two-sided *P* value <0.05 indicated statistical significance.

## 3. Results

### 3.1. Literature Screening Results

**Figure 1:**
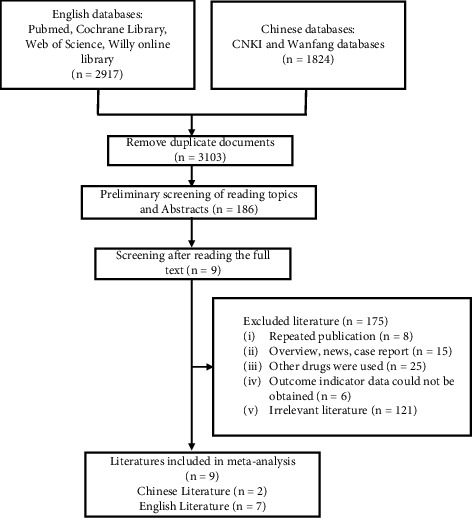
Document screening and inclusion process.

**Figure 2:**
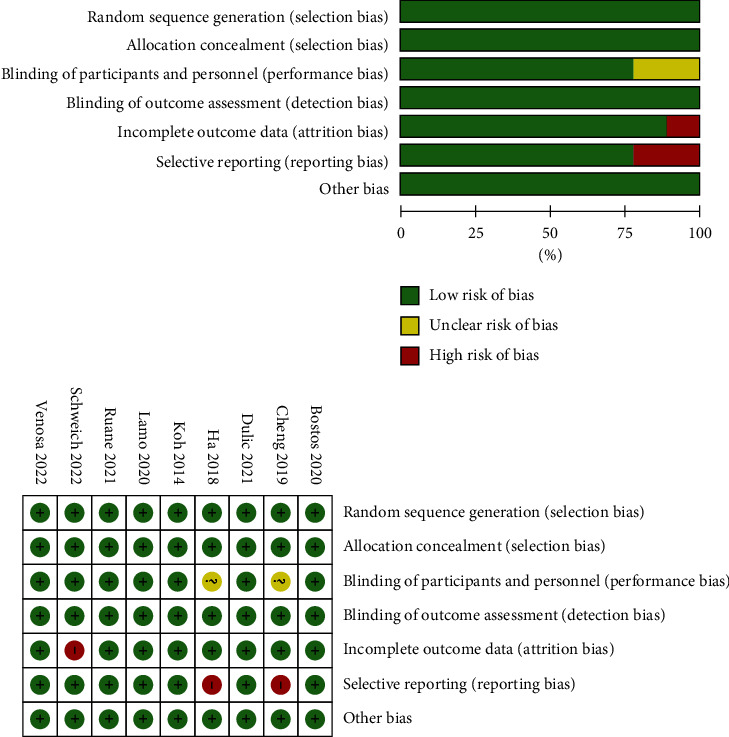
Literature risk-of-bias evaluation.

A total of 4741 articles related to KOA treatment by PRP combined with MSCs were found through Chinese and English data retrieval, of which 2917 were in English and 1824 in Chinese. After removing the duplicate publications, there were 3103 articles left. The remaining literature was read with titles and abstracts, and 2917 papers inconsistent with the eligibility criteria were removed. After obtaining and reading the full text, 8 papers were excluded for duplication or repeated entries, 15 excluded for nonoriginal articles, and 25 excluded for the incorporation of additional treatments in the intervention group. In addition, data with regard to outcome indicators could not be obtained, and 6 were not related to the theme of this article. Finally, 9 RCTs [3,5,11,12,18–22], including 7 English documents and 2 Chinese documents, were analyzed (Figure 1). The risk-of-bias assessment is shown in Figure 2.

### 3.2. Basic Characteristics of the Included Literature

The publication time of the 9 included RCTs ranged from 2014 to 2022, of which two were from China [3,11], two were from the United States [5, 19], and two were from Brazil [18, 21], as well as Serbia [12], Spain [20],and Italy, respectively [22], as shown in Table 1. A total of 480 KOA patients were included, including 277 patients in the interventional group and 203 patients in the control group. For the evaluation of literature quality, presence of one nonconformity was deemed to be of medium quality, two nonconformities were regarded as low quality, and more than three nonconformities were eliminated from this study. It was found that there were 6 high-quality documents, 1 medium-quality document, and 2 low-quality documents.

### 3.3. Combined Utility Value

#### 3.3.1. VAS Score

**Figure 3:**
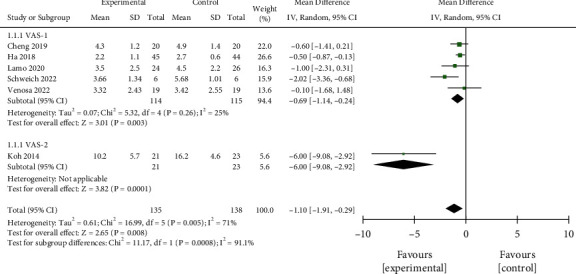
Comparison of VAS scores between the two groups.

**Figure 4:**
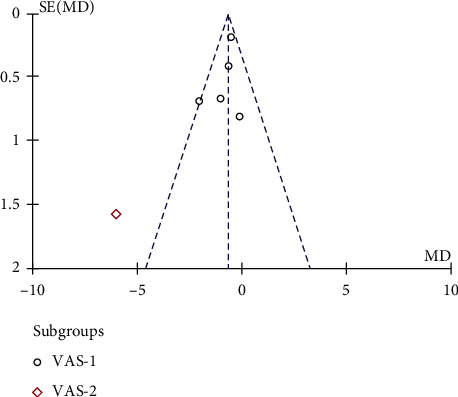
Funnel plot for the comparison of VAS scores between the two groups.

Six studies, including 135 cases in the interventional group and 138 cases in the control group, were analyzed. Significant heterogeneity was found (*P*=0.005, *I*^2^ = 71%), which was predominantly caused by the study of Koh et al. [19]. Thus, subgroup analysis was performed. The results showed that treatment with PRP combined with MSCs was associated with a significantly lower VAS score (MD = −1.10, 95% CI −1.91, −0.29, *P*=0.0008, Figure 3). Subgroup analysis found that MD = −0.69 (95% CI (−1.14, −0.24), *P*=0.003) in the VAS-1 group, while there was only 1 dataset in VAS-2 group, so pooled analysis was not performed. The results indicated that the pain relief in KOA patients treated with PRP combined with MSCs was more significant. The funnel plot showed that the points were symmetrically distributed on both sides, suggesting there was no obvious publication bias (Figure 4).

#### 3.3.2. KOOS

**Figure 5:**
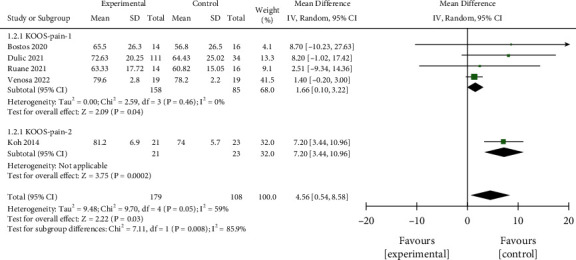
Comparison of forest chart of KOOS pain scores between the two groups.

**Figure 6:**
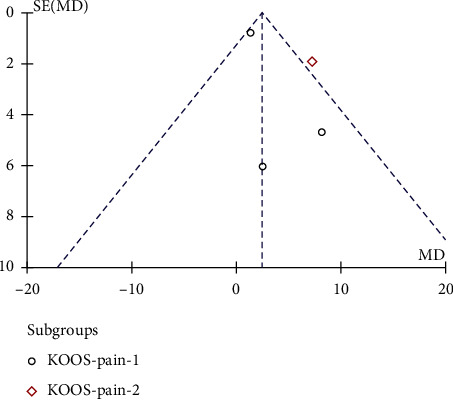
Funnel plot for the comparison of KOOS pain scores between the two groups.

Random-effect analysis of 5 studies that included 181 cases in the interventional group and 106 controls showed no significant difference between the two groups in terms of KOOS pain score (MD = 4.56, 95% CI 0.54,8.58, *P*=0.05, Figure 5) after treatment for 12 months. Significant interstudy heterogeneity (*P*=0.05, *I*^2^ = 59%) was noted to be predominantly contributed by the study by Koh et al. [19]. Subgroup analysis found that MD = 1.66 in KOOS-pain-1 group (95% CI (0.10, 3.22), *P*=0.04) and the difference was statistically significant. The KOOS-pain-2 group had only one dataset, and no combined analysis was performed. The funnel plot indicated the absence of obvious publication bias (Figure 6).

#### 3.3.3. KOOS Total Score

**Figure 7:**
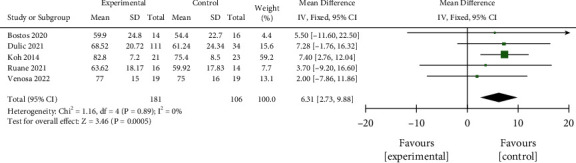
Comparison of total scores of two groups of KOOS.

**Figure 8:**
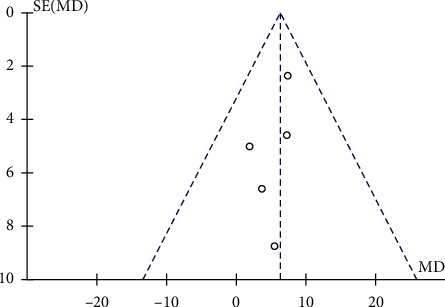
Funnel plot for the comparison of KOOS total scores between the two groups.

Fixed-effect model (*P*=0.89, *I*^2^ = 0%) analysis of 5 studies with 181 interventional cases and 106 controls found significantly increased KOOS total score in the interventional group as compared with the control group (MD = 6.31, 95% CI 2.73–9.88, *P*=0.0005, Figure 7) after treatment for 12 months. No significant publication bias was found (Figure 8).

#### 3.3.4. WOMAC Arthritis Index

**Figure 9:**
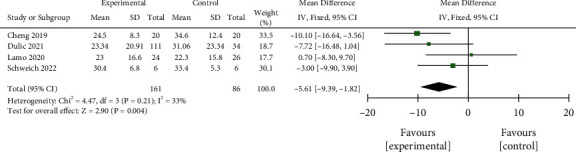
Comparison of two groups of WOMAC total score forest map.

**Figure 10:**
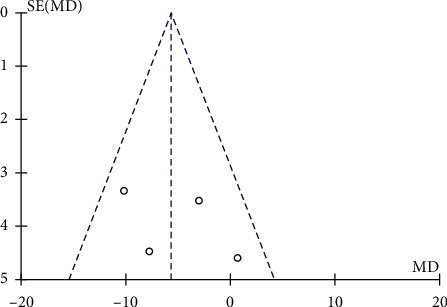
Funnel plot for the comparison of WOMAC total scores between the two groups.

Data of 161 interventional cases and 86 controls in 4 studies that employed WOMAC arthritis index as the outcome indicator were pooled using the fixed-effect model. We found a significantly decreased WOMAC arthritis index in the interventional group as compared with the control group after treatment for 12 months (MD = −5.61, 95% CI −9.39 to −1.82, *P*=0.004, Figure 9), suggesting enhanced knee function recovery in the former group. As shown in Figure 10, the funnel plot showed the absence of obvious publication bias.

#### 3.3.5. IKDC Score

**Figure 11:**
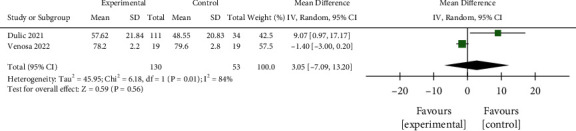
Comparison of IKDC scores between the two groups.

**Figure 12:**
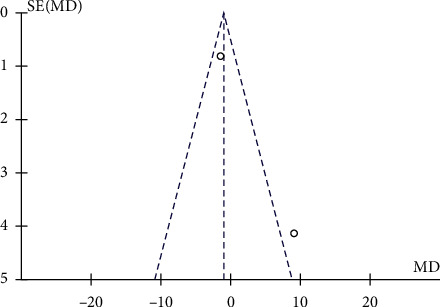
Funnel plot for the comparison of IKDC scores between the two groups.

Since there was significant interstudy heterogeneity (*P*=0.01, *I*^2^ = 84%), the random-effect model was employed to analyze the 2 studies that included 130 patients in the interventional group and 53 in the control group. As shown in Figure 11, no significant differences regarding the IKDC score after 12 months treatment were noted (MD = 3.05, 95% CI −7.09 to −13.20, *P*=0.56). Publication bias evaluation showed that points were distributed on both sides, but one item was outside the confidence interval. Egger's test (Figure 12) showed there was no obvious publication bias (*P*=1.00 > 0.05).

## 4. Discussion

Due to the increase in people's life expectancy and sports activities, the prevalence of KOA has increased significantly [23]. Intra-articular injections of biological agents have aroused great interest as a form of nonsurgical treatment. However, the superiority of different biological agents is still controversial. PRP and MSC have been showing promising results in the treatment of KOA for their regenerative potential that are expected to control disease progression [2, 24]. It is well known that the inflammatory process of KOA is directly related to pain, edema, redness, and joint stiffness [25–27]. These symptoms are associated with the reduction of daily activity and decline of life quality. Some studies have found that application of MSC combined with PRP for intra-articular injection could significantly improve the VAS score, KOOS pain score and total score, WOMAC arthritis index, and IKDC in KOA patients [3, 12, 20, 21].

This meta-analysis showed that after 12 months of treatment with PRP combined with MSCs, there were no significant differences in terms of KOOS pain score and IKDC score between the 2 groups. In contrast, significant differences in the VAS score, KOOS total score, and the total score of WOMAC arthritis index were observed. PRP combined with MSCs could significantly reduce knee pain in KOA patients, thus improving their quality of life. In addition, this treatment method is safe and there is no clinical evidence showing MSC alone or in combination with PRP may increase the risk of malignancy or immune disorders [12, 21, 28]. A previous study even reported that the treatment group was associated with a lower incidence of tumors than the general population [29].

In this study, the result showed high interstudy heterogeneity in some analyses, which may be related to the fact that there was no relatively unified standard for the treatment with PRP combined with MSCs. Meanwhile, the MSCs were derived from various sources, including adipose tissue and the bone marrow. In addition, the practice of PRP administration also varies among different regions or countries.

This study suffers from several limitations: (1) although both the Chinese and English keywords were used to search the literature, there is no guarantee that there will be no omissions; (2) the number of reports on the treatment of KOA by PRP combined with MSCs was insufficient, so only 9 papers were included in this study; (3) the sample size included in the literature was only 480 cases, and there were certain differences in terms of the source of MSCs and the injection method of PRP; (4) the 9 papers included were from six countries; although the literature quality was acceptable, there might be differences in ethnic and geographical factors; (5) the studies included in the meta-analysis were controlled for different confounding factors, and the combined effect size may be biased to a certain extent. It is expected that more results will be added in follow-up studies to make the conclusions more reliable.

In conclusion, intra-articular injection of PRP combined with MSCs in treating KOA can significantly alleviate patients' knee pain and improve knee function within one year. Compared with the control group, it shows significantly improved clinical effects. However, due to the limitation of this study, the conclusion still needs to be verified in prospective, multicenter studies with sufficient sample size.

## Figures and Tables

**Table 1:** Basic data of patients in 9 papers.

Included literature	Country	Study type	Number of patients		Age (average)		Sex ratio (male/female)		BMI (average)	Arthritis grade I/II/III/IV		Interventions	Follow-up time	Outcome indicators
			T	C	T	C	T	C	T/C	T	C			
Bostos et al. [18]	Brazil	RCT	14	16	60.8	55.7	9/5	6/10	28.9/30.6	1/3/6/4	1/7/5/3	PRP-MSC	1, 12	(2) (3)
Cheng[3]	China	RCT	20	20	54.6	52.9	8/15	9/11	—	8/8/4/0	7/8/5/0	PRP-MSC	1, 3, 6	(1) (4)
Dulic et al. [12]	Serbia	RCT	111	34	56.9	58.8	57/54	15/19	28.6/28.47	0/49/46/16	0/12/12/10	PRP-MSC	1, 3, 6, 9, 12	(2) (3) (4) (5)
Ha et al. [11]	China	RCT	45	44	56.8	55.6	15/30	14/30	25.5/25.4	—	—	PRP-MSC	1, 3, 6, 12	(1)
Koh et al. [19]	USA	RCT	21	23	54.2	52.3	5/16	6/17	25.7/24.7	—	—	PRP-MSC	1, 12	(1) (2) (3)
Lamo-Espinosa et al. [20]	Spain	RCT	24	26	56	54.6	17/7	16/10	27/25.3	5/5/2/12	0/8/5/13	PRP-BM/MSC	1, 3, 6, 12	(1) (4)
Schweich-Adami et al. [21]	Brazil	RCT	6	6	52.66	48	3/3	4/2	31.08/27.3	0/2/3/1	0/2/4/0	PRP-AD/MSC	1, 6	(1) (4)
Ruane et al. [5]	USA	RCT	17	15	58.06	58.60	9/8	10/5	29.19/29.21	5/6/6/0	2/8/5/0	PRP-MSC	1, 3, 6, 12	(2) (3)
Venosa et al. [22]	Italy	RCT	19	19	55.8	56.4	9/10	12/7	25.8/26.2	0/0/0/19	0/0/0/19	PRP-AD/MSC	1, 3, 6, 12	(1) (2) (3) (5)

Note: RCT, randomized controlled trial; *P*, experimental group; *C*, control group. Outcome measures: (1) visual analogue scale (VAS) score, (2) knee injury and osteoarthritis outcome score (KOOS), (3) total KOOS score, (4) total Western Ontario and McMaster Universities (WOMAC) score; and (5) International Knee Documentation Committee (IKDC) score.

## Data Availability

The data used and analyzed during the current study are available from the corresponding author.
